# The association of dietary inflammatory index with auditory and vestibular disorders in Korean adults: Differential links to peripheral vertigo, hearing loss and tinnitus

**DOI:** 10.1016/j.clinsp.2026.101045

**Published:** 2026-07-08

**Authors:** Nan-Ren Sun, Sheng-Yuan Fang, Jiao-Jiao Chen, Qingyang Wang, Zhi-Min Tan

**Affiliations:** a Shandong University of Traditional Chinese Medicine, Jinan, Shandong Province, China; b Anhui Provincial Hospital of Traditional Chinese Medicine, Hefei, Anhui Province, China; c Guangzhou Medical University, Dongguan, Guangdong Province, China; d Department of Otorhinolaryngology, the Affiliated Hospital, Shandong University of Traditional Chinese Medicine, Jinan, Shandong Province, China

**Keywords:** Otorhinolaryngology, Hearing loss, Tinnitus, Peripheral vertigo, Anti-inflammatory diet, Dietary inflammatory index (dii), Epidemiology, Knhanes

## Abstract

**Background:**

The auditory and vestibular systems are crucial for human perception, spatial orientation, and balance control. Dysfunction in these systems is a significant public health issue. This study explores the relationship between the Dietary Inflammatory Index (DII) and auditory-vestibular disorders such as hearing loss, tinnitus, and Peripheral Vertigo (PV).

**Methods:**

The authors used data from the 2020–2023 Korea National Health and Nutrition Examination Survey (KNHANES) to assess the associations between DII and hearing loss, tinnitus, and PV in Korean adults. A cross-sectional design was employed, with DII calculated based on dietary recall.

**Results:**

The study found significant associations between DII and moderate-to-severe hearing loss and PV. Specifically, higher DII scores were associated with an increased odds of hearing loss and PV. Subgroup analyses showed that women and older adults exhibited a stronger association between DII and hearing loss.

**Conclusion:**

The present findings suggest an association between higher DII and both hearing loss and PV. However, the cross-sectional design limits causal inference. Future longitudinal studies are needed to confirm the role of dietary inflammation in auditory and vestibular dysfunction.

## Highlights

• Higher DII scores were significantly associated with peripheral vertigo and hearing loss.
• Dietary inflammation showed a stronger link to moderate-to-severe hearing loss.
• Women and older adults were more vulnerable to the effects of dietary inflammation.
• Tinnitus showed no significant association with DII in adjusted models.
• Restricted cubic splines revealed nonlinear relationships between DII and outcomes. Findings highlight diet-related inflammation as a potential target for sensory health.

## Background

The auditory and vestibular systems are pivotal to human perception, spatial orientation, and balance control; dysfunction in these systems has become a significant public health concern that substantially degrades quality of life.^1^, ^2^, ^3^ Hearing loss, tinnitus, and Peripheral Vertigo (PV) are the most prevalent auditory- and vestibular-related disorders encountered in clinical practice.^4^^,^^5^ Prior studies in Korea indicate that auditory-vestibular conditions ‒ hearing loss, tinnitus, and PV ‒ are highly prevalent, with the burden particularly pronounced in older adults.^6^, ^7^, ^8^ Despite their distinct clinical presentations, hearing loss, tinnitus, and PV commonly lead to impaired balance, cognitive decline, and restrictions in physical activity, thereby imposing substantial burdens on quality of life, healthcare utilization, and the broader economy.^9^, ^10^, ^11^

Diet has garnered increasing attention in recent years as a modifiable source of inflammation. Existing evidence indicates that anti-inflammatory dietary patterns ‒ exemplified by the Mediterranean diet ‒ are associated with chronic dizziness/imbalance and with reduced risk of cognitive decline.^12^^,^^13^ DII is a composite metric derived from extensive epidemiologic and experimental evidence and widely used to quantify the inflammatory potential of an individual’s diet. The DII is computed by integrating the direction and magnitude of multiple dietary components’ effects on inflammatory biomarkers ‒ such as C-reactive protein, interleukin-6, and tumor necrosis factor-α ‒ with higher scores indicating greater pro-inflammatory potential and lower scores reflecting stronger anti-inflammatory characteristics.^14^^,^^15^

Nevertheless, evidence linking the DII to auditory and vestibular disorders remains limited ‒ particularly at the population level for differential associations of dietary inflammation with hearing loss, tinnitus, and PV ‒ and distinct conditions may exhibit heterogeneous sensitivity to inflammatory exposure.^16^ Accordingly, investigating diet-sensory disorder relationships through an inflammatory lens may clarify disease-specific differences in inflammatory susceptibility. Building on this premise, the authors leverage nationally representative data from the 2020–2023 Korea National Health and Nutrition Examination Survey (KNHANES) to systematically assess associations between DII and hearing loss, tinnitus, and PV in Korean adults, and to further examine disorder-specific heterogeneity in these associations. This study aims to elucidate potential pathways by which dietary inflammatory potential may be related to auditory and vestibular dysfunction and to provide epidemiologic evidence to guide dietary interventions and sensory health promotion.

## Method

### Data source and study population

This study used data from the Korea National Health and Nutrition Examination Survey (KNHANES) collected between 2020 and 2023, covering Cycle VIII (2020–2021) and Cycle IX (2022–2023). KNHANES is administered by the Korea Disease Control and Prevention Agency (KDCA) to monitor health risk factors and trends in chronic disease among Korean residents, and comprises a health examination, health interview, and nutrition survey conducted by trained medical personnel and interviewers. The survey protocol was approved by an institutional ethics committee, and all participants provided written informed consent. Detailed information on the survey design and instruments is available on the KNHANES website (https://knhanes.kdca.go.kr/). This cross-sectional study was reported in accordance with the Strengthening the Reporting of Observational Studies in Epidemiology (STROBE) guidelines.

**Fig. 1:**
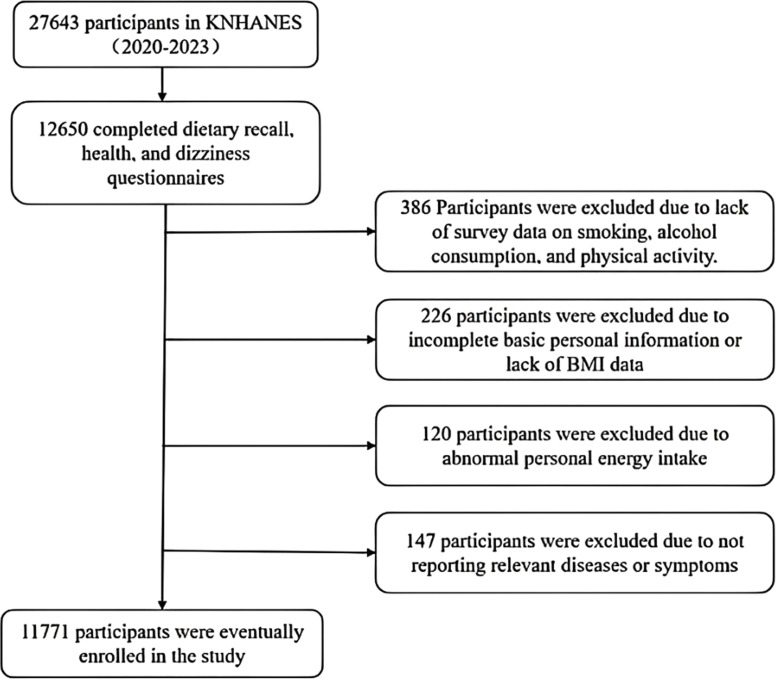
Flowchart of participant inclusion and exclusion in KNHANES (2020–2023).

From 2020 to 2023, 27,643 individuals were sampled, of whom 12,650 completed the health examination, disease-related questionnaires, and nutrition survey. The authors excluded participants who lacked survey data on smoking, alcohol consumption, or physical activity (n = 386); lacked basic demographic information or Body Mass Index (BMI) measurements (n = 226); reported implausible daily energy intake (< 500 or > 6500 kcal; n = 120); or did not report other diseases or symptoms (hypertension, diabetes, psychological stress; n = 147). The final analytic sample comprised 11,771 participants (Fig. 1). The authors compared the included and excluded participants on key demographic characteristics and found that they were generally similar, suggesting that the exclusion process was unlikely to substantially affect the overall interpretation of the findings.

### DII calculation

In this study, DII was computed using the established standard model. Specifically, intakes of 45 dietary nutrients were first standardized to global reference means to obtain Z-scores, which were then multiplied by the corresponding inflammatory effect weights for each component. The weighted values were summed to yield each participant’s overall DII score. Higher DII values indicate greater pro-inflammatory potential, whereas lower values reflect stronger anti-inflammatory potential.

Dietary intake data were obtained from KNHANES via a one-hour interviewer-administered dietary recall that captured participants’ food consumption over the preceding 48-hours; individual daily nutrient intakes were calculated with the 10th edition of the Korean Food Composition Table issued by the Rural Development Administration.^17^ Because several nutrients were missing in the 2020–2023 KNHANES files, the authors computed DII scores using 23 nutrients with complete records, and the detailed list of these components is provided in Supplementary Table S1. Prior studies indicate that a modified DII including ≥ 20 key nutrients can still validly reflect the inflammatory potential of diet and has acceptable applicability and interpretability in epidemiologic research.^18^

### Hearing loss, tinnitus, and peripheral vertigo definitions

Hearing loss was determined based on pure-tone audiometry results provided by the KNHANES. The analysis focused on participants aged ≥ 40 years who had completed bilateral pure-tone audiometry with complete data. For each participant, the hearing thresholds were measured at 500, 1000, 2000, and 4000 Hz for both ears (measured in Decibels [dB]). The Pure-Tone Average (PTA) was then calculated as the average of the thresholds at these four frequencies: PTA=(500Hz+1000Hz+2000Hz+4000Hz)/4.

This method provided a standardized measure of hearing sensitivity across the most commonly tested frequencies.

For participants with “no response” recorded at certain frequencies, the hearing thresholds were substituted with the maximum measurable value for each frequency band (0.5–4 kHz: −10 to 100 dB; 8 kHz: −10 to 90 dB) and included in the calculation. Additionally, to ensure the accuracy of the measurements, the hearing tests were conducted in soundproof rooms meeting ANSI-ASA S3.1 standards. If the environmental noise level exceeded the allowable limits on the testing day, the participant’s hearing results for that day were excluded.

The degree of hearing loss was defined as follows: A Pure-Tone Average (PTA) ≥ 26 dB was considered all hearing loss. If the PTA was ≥ 26 dB but < 41 dB, it was classified as mild hearing loss. If the PTA for either ear was ≥ 41 dB, it was categorized as moderate-to-severe hearing loss.

In KNHANES, tinnitus- and dizziness-related symptoms are collected via standardized questionnaires administered during the health interview; therefore, tinnitus and PV were operationalized based on participants’ responses to these items.

Tinnitus was determined based on responses to a specific tinnitus-related questionnaire. Participants were asked two questions: 1) “In the past 12-months, have you heard persistent sounds in your ears (such as buzzing, ringing, or mechanical noises) for >5-minutes without an external sound source?”; 2) “Has this sound persisted for >6-months?”.

If the participant answered “yes” to both questions, they were classified as having tinnitus. This criterion was used to identify participants with subjective tinnitus symptoms that had occurred and persisted for at least 6-months within the past year.

PV was determined based on responses to a dizziness-related questionnaire. Participants were classified as having PV if they met the following criteria: 1) “In the past 12-months, have you experienced dizziness or imbalance?”; 2) “Has the dizziness or imbalance occurred more than twice?”; 3) “In the past three months, have you felt difficulty maintaining posture while standing?”; 4) “In the past three months, have you experienced difficulty walking?”; 5) “In the past three months, have you experienced persistent dizziness?”; 6) “In the past three months, have you repeatedly fallen?”. PV was diagnosed only when the first two questions were answered as “yes”, and the latter four questions were answered as “no”.

### Covariate definitions

In this study, the following covariates were controlled for: demographic characteristics (age, sex, residential area, marital status, education level, occupation), lifestyle factors (alcohol consumption, smoking, walking exercise, resistance training), physiological and psychological health status (diabetes, hypertension, perceived stress level), and Body Mass Index (BMI). Household income was categorized into quartiles according to Korean standards. Physical activity levels were classified based on the number of exercise days per week as “none”, “low” (1–4 days), or “high” (≥ 5-days). Diabetes and hypertension were defined based on physician diagnosis, while perceived stress was based on self-report. BMI was categorized according to Asian standards as underweight (< 18.5 kg/m^2^), normal weight (18.5–24.9 kg/m^2^), and overweight (≥ 25 kg/m^2^).

### Statistical analysis

Participants were categorized into quartiles of DII (T1–T4) according to the distribution of DII scores in the study population, with T1 (lowest quartile) serving as the reference group. Descriptive statistics of basic characteristics were weighted: continuous variables were presented as weighted means with Standard Errors (SE), and categorical variables as weighted percentages with their SE. Differences in continuous variables were assessed using the weighted Kruskal-Wallis test, while categorical variables were analyzed using the Rao-Scott χ^2^ test. To evaluate the association between DII and hearing loss, tinnitus, and PV, weighted multinomial logistic regression models were constructed, reporting Odds Ratios (OR) and their 95% Confidence Intervals (95% CI). Model 1 adjusted for age and sex. Model 2 further adjusted for education and other sociodemographic characteristics. Model 3 included additional covariates, such as lifestyle factors, BMI, and relevant comorbidities. To explore the moderating effects of sex and age, stratified analyses were conducted based on Model 3, assessing the association of DII across different sex and age groups. The log(OR) and SE for the same DII levels were calculated for each stratification, and Wald tests were used to assess the statistical significance of interaction terms. To analyze the potential nonlinear relationship between DII and odds of the outcomes, Restricted Cubic Splines (RCS) models were used. The knots were placed at the 10th, 50th, and 90th percentiles of the DII distribution, with adjustments for sociodemographic covariates in the model.

All statistical analyses were performed using SAS 9.4 software (SAS Institute Inc., Cary, NC, USA). Sample weights were incorporated into the analysis to account for the complex sampling design. These weights were calculated by KNHANES based on individual sampling probabilities, non-response adjustments, and post-stratification coefficients to ensure the representativeness of the results for the national adult population. A significance level of two-sided p < 0.05 was used.

## Result

### Analysis of the association between DII and lifestyle, socioeconomic status

Table 1 (T1) presents the characteristics of study participants according to the DII quartiles. The data indicate significant differences across the DII groups in terms of gender, education level, household income, smoking, alcohol consumption, physical activity, and perceived stress levels. These variables showed varying distributions within each quartile, suggesting that lifestyle and socioeconomic factors are associated with dietary inflammatory potential, as reflected by DII scores.

**Table 1:** Baseline characteristics of participants across quartiles of the Dietary Inflammatory Index (DII).

**DII group / Covariate**	**T1** **(n=2942)**	**T2** **(n=2943)**	**T3** **(n=2943)**	**T4** **(n=2943)**	**p-value**
**Age (years)**	58.10 ± 0.23	57.92 ± 0.23	57.81 ± 0.24	57.73 ± 0.25	0.8289
**Sex**					**<0.0001**
Men	59.1 (1.0)	52.7 (1.1)	47.6 (1.1)	34.9 (1.1)	
Women	40.9 (1.0)	47.3 (1.1)	52.4 (1.1)	65.1 (1.1)	
**Residential area**					0.4628
Urban	82.5 (0.8)	82.4 (0.8)	82.2 (0.7)	81.0 (0.8)	
Rural	17.5 (0.8)	17.6 (0,8)	17.8 (0.7)	19.0 (0.8)	
**Education**					**<0.0001**
Below college	55.8 (1.1)	59.5 (1.1)	62.1 (1.1)	66.7 (1.1)	
College or above	44.2 (1.1)	40.5 (1.1)	38.9 (1.1)	33.3 (1.1)	
**Occupation**					**0.0019**
Non-manual labor	74.4 (0.9)	71.68 (1.0)	74.1 (1.0)	76.8 (0.9)	
Manual labor	25.6 (0.9)	28.32 (1.0)	25.9 (1.0)	23.2 (0.9)	
**Household income**					**<0.0001**
Q1	19.8 (0.9)	21.4 (0.90)	23.7 (0.9)	28.7 (1.0)	
Q2	23.0 (0.91)	24.6 (1.0)	26.3 (1.0)	25.5 (0.9)	
Q3	27.0 (0.97)	25.1 (1.0)	25.1 (0.9)	23.9 (0.9)	
Q4	30.2 (1.00)	28.9 (1.0)	24.9 (0.9)	21.9 (0.9)	
**Drinking status**					**<0.0001**
No	90.9 (0.6)	90.1 (0.6)	89.0 (0.6)	86.9 (0.7)	
Yes	9.1 (0.6)	9.9 (0.6)	11.0 (0.6)	13.1 (0.7)	
**Smoking status**					**<0.0001**
Smoke Now	15.8 (0.8)	15.2 (0.8)	17.3 (0.9)	16.9 (0.8)	
Smoke in the past	34.4 (1.1)	29.6 (1.0)	25.8 (1.0)	18.1 (0.8)	
NO	49.7 (1.1)	55.3 (1.1)	57.0 (1.1)	65.0 (1.0)	
**Weight training**					**<0.0001**
No	67.0 (1.0)	71.8 (1.0)	75.6 (0.9)	81.7 (0.9)	
4 times or less	19.2 (1.0)	16.6 (0.8)	15.8 (0.8)	12.4 (0.7)	
5 times or more	13.8 (0.8)	11.7 (0.7)	8.6 (0.6)	5.9 (0.5)	
**Walking training**					**<0.0001**
No	13.0 (0.7)	15.6 (0.8)	16.24 (0.8)	21.79 (0.9)	
4 times or less	36.8 (1.06)	36.7 (1.1)	38.33 (1.1)	37.12 (1.0)	
5 times or more	50.1 (1.09)	47.8 (1.1)	45.43 (1.1)	41.09 (1.1)	
**BMI**					0.2998
Underweight	2.6 (0.3)	2.6 (0.4)	3.1 (0.4)	3.4 (0.4)	
Normal weight	58.5 (1.1)	58.0 (1.1)	59.1 (1.1)	60.3 (1.1)	
Overweight	38.9 (1.1)	39.4 (1.1)	37.8 (1.1)	36.3 (1.0)	
**Psychological stress**					**0.0163**
Severe	2.75 (0.3)	3.1 (0.4)	3.4 (0.4)	4.9 (0.5)	
Moderate	17.9 (0.8)	19.1 (0.9)	19.3 (0.9)	19.5 (0.85)	
Mild	60.7 (1.1)	60.4 (1.1)	60.1 (1.1)	60.4 (1.1)	
No	18.7 (0.8)	17.4 (0.8)	17.2 (0.8)	15.3 (0.8)	
**History of hypertension**					0.1966
No	60.7 (1.0)	69.4 (1.0)	67.4 (1.0)	67.4 (1.0)	
Yes	30.4 (1.0)	30.6 (1.0)	32.6 (1.0)	32.6 (1.0)	
**History of diabetes**					0.8085
No	86.5 (0.7)	85.7 (0.7)	86.4 (0.73)	86.7 (0.7)	
Yes	13.5 (0.7)	14.3 (0.7)	13.6 (0.73)	13.3 (0.7)	

Note: All descriptive statistics were calculated using KNHANES survey weights. Continuous variables are presented as weighted means with Standard Errors (SE), and categorical variables as weighted percentages with SE. n denotes the unweighted number of participants. Bold values indicate statistical significance (p < 0.05).

### Association between DII and PV, hearing loss, and tinnitus

Table 2 (T2) presents the cross-sectional associations between DII and PV, hearing loss, and tinnitus. In Model 1, significant differences were observed between the highest DII quartile (T4) and the reference group (T1) in the PV group, overall hearing loss group, and the moderate-to-severe hearing loss group, indicating a potential positive correlation between higher DII scores and the odds of PV and hearing loss. After adjusting for basic lifestyle variables and further incorporating multivariable adjustments, this positive association remained significant, suggesting that DII continues to be associated with an increased odds of both PV and hearing loss, even after controlling for other influencing factors.

**Table 2:** Associations of dietary inflammatory index quartiles with hearing loss, PV, and tinnitus: multivariable logistic regression models.

	**DII**						
	**T1**	**T2**		**T3**		**T4**	
	**ORs**	**ORs (95% CIs)**	**p-value**	**ORs (95% CIs)**	**p-value**	**ORs (95% CIs)**	**p-value**
**Model 1A**	1.0 (ref)	1.05 (0.90‒1.23)	0.5363	1.04 (0.90‒1.21)	0.6446	**1.23 (1.06‒1.43)**	**0.0070**
**Model 2A**	1.0 (ref)	1.05 (0.90‒1.23)	0.5383	1.03 (0.88‒1.20)	0.7255	**1.20 (1.04‒1.40)**	**0.0147**
**Model 3A**	1.0 (ref)	1.04 (0.89‒1.21)	0.6111	1.01 (0.87‒1.18)	0.8846	**1.18 (1.02‒1.38)**	**0.0313**
**Model 1B**	1.0 (ref)	1.03 (0.89‒1.20)	0.7072	**1.18 (1.01‒1.38)**	**0.0331**	**1.36 (1.17‒1.59)**	**<0.0001**
**Model 2B**	1.0 (ref)	1.00 (0.86‒1.17)	0.9668	1.13 (0.97‒1.32)	0.1175	**1.26 (1.08‒1.48)**	**0.0031**
**Model 3B**	1.0 (ref)	0.99 (0.85‒1.15)	0.8924	1.10 (0.94‒1.28)	0.2338	**1.20 (1.03‒1.41)**	**0.0185**
**Model 1C**	1.0 (ref)	0.97 (0.84‒1.11)	0.6627	0.97 (0.84‒1.13)	0.7124	1.04 (0.90‒1.20)	0.6246
**Model 2C**	1.0 (ref)	0.95 (0.82‒1.09)	0.4449	0.94 (0.81‒1.14)	0.4182	0.99 (0.85‒1.14)	0.8458
**Model 3C**	1.0 (ref)	0.94 (0.81‒1.08)	0.3623	0.92 (0.79‒1.07)	0.2729	0.95 (0.82‒1.10)	0.5328
**Model 1D**	1.0 (ref)	1.04 (0.86‒1.25)	0.6907	**1.23 (1.02‒1.35)**	**0.0287**	**1.41 (1.17‒1.69)**	**0.0002**
**Model 2D**	1.0 (ref)	1.00 (0.83‒1.21)	0.9663	1.16 (0.96‒1.39)	0.1237	**1.29 (1.08‒1.55)**	**0.0061**
**Model 3D**	1.0 (ref)	0.99 (0.82‒1.19)	0.9172	1.12 (0.94‒1.36)	0.2010	**1.24 (1.02‒1.49)**	**0.0274**
**Model 1E**	1.0 (ref)	0.96 (0.78‒1.18)	0.6933	0.85 (0.69‒1.06)	0.1480	0.92 (0.73‒1.15)	0.4459
**Model 2E**	1.0 (ref)	0.95 (0.77‒1.18)	0.6600	0.84 (0.68‒1.05)	0.1213	0.90 (0.72‒1.13)	0.3543
**Model 3E**	1.0 (ref)	0.95 (0.77‒1.18)	0.6525	0.84 (0.68‒1.05)	0.1258	0.90 (0.71‒1.13)	0.3590

Note: Model 1 is age and sex adjusted. Model 2 is sex, age, marital status, Residential area, Education, Occupation and Household income adjusted. Model 3 was multivariate adjusted. (A) PV; (B) Overall hearing loss; (C) Mild hearing loss; (D) Moderate or worse hearing loss; (E) Tinnitus.

### Subgroup analysis

Table 3 presents the results of the subgroup analysis, where the authors found significant associations between PV, overall hearing loss, and moderate-to-severe hearing loss in females and older age groups, while no significant relationships were observed in the younger age or male groups. Analysis of subgroup interaction effects revealed that sex stratification had a significant influence on the association between DII and both overall hearing loss and moderate-to-severe hearing loss. However, no significant interaction effects were found in the other subgroup analyses. Further analysis showed that higher education levels, non-drinking, and moderate physical activity (including walking or limited resistance training) were protective factors for hearing loss in various subgroups. In contrast, comorbidities such as hypertension and diabetes were identified as significantly associated with overall hearing loss. Additionally, smoking was found to be a potential risk factor for hearing loss in the male and middle-aged subgroups. Regarding PV, the results indicated that psychological stress, hypertension, and diabetes were associated with PV across different populations. Detailed subgroup covariate estimates are shown in Supplementary Tables S2‒S3.

**Table 3:** Sex- and age-stratified subgroup analysis of the associations between DII and PV, overall hearing loss, and moderate or greater hearing loss.

**Outcome, OR (95% CI) / Sex group**	**Peripheral vertigo**		**Moderate-to-severe hearing loss**		**Overall hearing loss**	
	**Men**	**Women**	**Men**	**Women**	**Men**	**Women**
DII [G2 vs. G1]	0.93 (0.74‒1.17)	1.06 (0.86‒1.30)	0.96 (0.76–1.22)	1.13 (0.84–1.51)	0.97 (0.81‒1.17)	0.95 (0.78‒1.17)
DII [G3 vs. G1]	0.93 (0.73‒1.19)	1.08 (0.88‒1.32)	1.18 (0.92–1.52)	1.04 (0.79–1.38)	1.10 (0.91‒1.33)	0.96 (0.79‒1.16)
DII [G4 vs. G1]	1.02 (0.79‒1.33)	**1.29 (1.03‒1.43)**	1.08 (0.82–1.42)	**1.45 (1.11–1.89)**	1.01 (0.81‒1.25)	**1.21 (1.00‒1.45)**
**Age group**	**Low Age (≤ 60)**	**High Age (> 60)**	**Low Age (≤ 60)**	**High Age (> 60)**	**Low Age (≤ 60)**	**High Age (> 60)**
DII [G2 vs. G1]	1.03 (0.83–1.28)	0.93 (0.75–1.15)	0.96 (0.63–1.46)	1.12 (0.92–1.35)	0.95 (0.76‒1.18)	1.12 (0.93‒1.34)
DII [G3 vs. G1]	1.03 (0.83–1.28)	0.97 (0.78–1.20)	1.11 (0.73–1.68)	**1.22 (1.01–1.48)**	1.01 (0.81‒1.27)	**1.21 (1.01‒1.44)**
DII [G4 vs. G1]	1.11 (0.90–1.38)	**1.28 (1.04–1.58)**	1.12 (0.75–1.67)	**1.52 (1.25–1.85)**	1.02 (0.81‒1.28)	**1.49 (1.23‒1.81)**

Note: In subgroup analyses, participants were categorized into four groups (G1–G4) based on subgroup-specific quartiles of DII (G1: lowest quartile; G4: highest quartile), with G1 as the reference. Residential area, occupation, education, BMI category, alcohol consumption, smoking status, physical activity, psychological stress, hypertension, and diabetes were modeled as categorical variables; household income was modeled as a continuous variable. Bold indicates p < 0.05.

Interaction effects (sex): PV (p = 0.64); overall hearing loss (p = 0.03); Moderate-to-severe hearing loss (p = 0.01).

Interaction effects (age): PV (p = 0.44); overall hearing loss (p = 0.06); Moderate-to-severe hearing loss (p = 0.53).

### RCS analysis of DII and its association with PV, hearing loss, and tinnitus

**Fig. 2:**
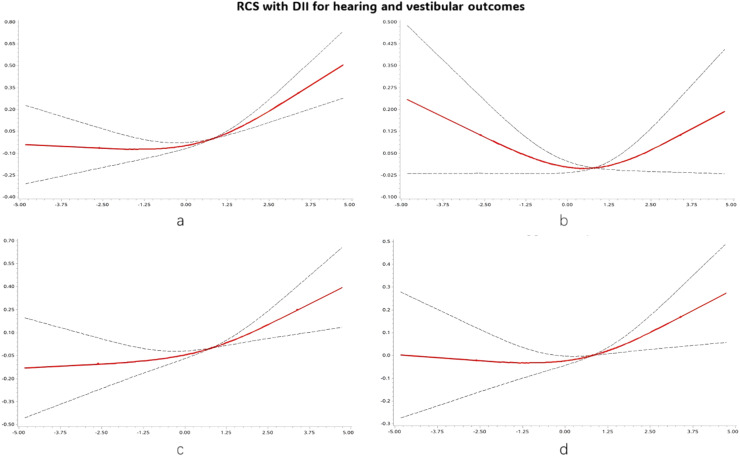
RCS analysis of the association between the Dietary Inflammatory Index (DII) and (a) Overall hearing loss, (b) Mild hearing loss, (c) Moderate-to-severe hearing loss, and (d) Peripheral Vertigo (PV). Note: The RCS models were fitted with covariates adjusted according to Model 3. The x-axis represents DII as a continuous variable. The y-axis represents the adjusted log Odds Ratio (log[OR]) with 95% Confidence Intervals (95% CIs), expressed relative to the reference DII value log[OR] = 0. The solid line indicates the estimated adjusted log(OR) across the range of DII, and the shaded area denotes the 95% CI.

In Fig. 2, the results of the Restricted Cubic Splines (RCS) analysis show that the association between DII and auditory-vestibular outcomes varied across different models. In the overall hearing loss model (Model 2a), both the overall association between DII and the outcome (p < 0.0001) and the nonlinear component (p = 0.0100) were statistically significant. In the mild hearing loss model (Model 2b), the overall association between DII and the outcome did not reach statistical significance (p = 0.1213), although the nonlinear component was significant (p = 0.0404). In the moderate to severe hearing loss model (Model 2c), the overall association between DII and the outcome was significant (p = 0.0003), but the nonlinear component was not significant (p = 0.1595). For the PV model (Model 2d), the overall association between DII and the outcome was significant (p = 0.0138), though the nonlinear component did not reach statistical significance (p = 0.1334) (Fig. 2).

## Discussion

In this large, nationally representative study of Korean adults, higher DII was associated with greater odds of PV and moderate-to-severe hearing loss. Previous studies have confirmed a close association between chronic low-grade inflammation and hearing loss as well as vestibular dysfunction.^19^^,^^20^ Chronic low-grade inflammation, particularly mild systemic inflammation that persists over time, has been widely recognized as the underlying pathological basis for many diseases, including cardiovascular diseases, diabetes, and neurodegenerative disorders.^21^ Hearing loss and vestibular dysfunction have also been regarded as being closely associated with the cumulative effects of systemic inflammation.^22^^,^^23^ Systemic inflammatory mediators play a crucial role in the inner‑ear pathology; these mediators not only exacerbate damage to the cochlear microvasculature but also trigger oxidative stress responses within the inner ear, thereby impairing neural transmission and causing hearing decline.^24^ Research has demonstrated that inflammatory mediators such as Tumor Necrosis Factor‑α (TNF‑α) and Interleukin‑6 (IL‑6) can induce apoptosis in cells within the inner ear, and by increasing vascular permeability and triggering local immune responses, they directly impact cochlear and auditory‑nerve function.^25^

PV often co‑occurs with vestibular dysfunction, a phenomenon closely linked to inflammation‑mediated disturbances of inner‑ear blood flow and neural responsiveness.^26^ The vestibular system, as the core sensor for balance perception, possesses neural structures that are highly sensitive to inflammatory mediators. These mediators may alter the permeability of inner‑ear microvasculature and thereby damage vestibular nerves, ultimately triggering dizziness (Peripheral Vertigo, PV).^27^ Research has demonstrated that systemic inflammation impacts vestibular function through multiple mechanisms, including inducing endothelial dysfunction in inner‑ear microvasculature with subsequent local hypoperfusion; promoting elevated oxidative stress levels in inner‑ear fluids that damage vestibular hair cells; and activating the NF‑κB pathway, which up‑regulates inflammatory mediators such as TNF‑α and IL‑6, thereby triggering neuroinflammation in vestibular pathways and disrupting normal vestibular signal transmission.^28^

Systemic inflammation may disrupt the interplay between the cochlear and vestibular systems, leading to neuro‑vascular unit dysfunction by compromising microvascular integrity, altering blood flow regulation, and impairing coordinated neural‑vascular interactions.^29^

These mechanisms may act in concert, increasing the risk for common PV such as otolithiasis (Benign Paroxysmal Positional Vertigo, BPPV), Ménière’s disease, and vestibular neuritis.^30^

The DII is a composite dietary scoring system designed to reflect the systemic inflammatory potential induced by diet. Numerous studies have shown that a high dietary inflammatory load is closely associated with a variety of adverse health outcomes, thereby leading to the widespread endorsement of anti‑inflammatory dietary patterns for the prevention and management of chronic diseases such as cardiovascular disease and diabetes.^31^ The present study further demonstrates that DII is significantly associated with PV and moderate‑to‑severe hearing loss. Specifically, moderate‑to‑severe hearing loss exhibited a significant linear positive association with DII (p = 0.0003), suggesting that higher DII is associated with higher odds of moderate-to-severe hearing loss. The overall association between DII and PV was also significant (p = 0.0138). However, the associations between DII and tinnitus or mild hearing loss were not statistically significant. Previous research has suggested that, in the pathophysiology of Ménière’s disease, cochlear and vestibular damage may occur in a dissociated pattern.^32^ Consistent with this concept, the authors observed differential associations of DII with tinnitus, hearing loss, and PV, suggesting that dietary inflammatory potential may influence auditory and vestibular outcomes through partially distinct pathways. Moreover, the stratified patterns indicate that the association between dietary inflammatory potential and hearing loss may be more evident at higher DII levels and in moderate-to-severe stages than in mild hearing loss. This pattern is hypothesis-generating and may be consistent with a stronger relationship between dietary inflammation and more advanced hearing impairment, warranting confirmation in longitudinal studies.^33^^,^^34^ In the stratified analyses, after excluding interactions that were not statistically significant, the authors observed a more pronounced association between dietary inflammatory load and hearing loss in women, suggesting that females may be more sensitive to systemic inflammation. This could be linked to immunological characteristics of women: previous studies have shown that females generally exhibit heightened immune responses and are more prone to vascular and neural damage under conditions of persistent low‑grade inflammation.^35^^,^^36^ In addition, women may exhibit a more pronounced response to dietary inflammatory burden in pathways related to metabolism and oxidative stress. A higher DII may promote the generation of Reactive Oxygen Species (ROS), impair mitochondrial function, and induce injury to vestibular neurons, and the relatively weaker antioxidant defenses in women may amplify these effects.^37^^,^^38^

Although this study reveals a significant association between DII and hearing loss as well as PV, there are several limitations. First, the study employed a cross‑sectional design, which precludes the ability to infer causal relationships between dietary inflammation and these disorders. Due to the lack of longitudinal data, it remains unclear whether higher dietary inflammatory potential precedes these outcomes or is associated with them cannot be determined. Second, the DII, tinnitus, and dizziness were based on questionnaire self-reports, which may be subject to recall/reporting bias and misclassification. In particular, peripheral vertigo was identified using questionnaire-based symptoms rather than physician-confirmed diagnosis; therefore, the authors’ definition should be interpreted as capturing individuals with symptoms consistent with peripheral vertigo, and some outcome misclassification may be unavoidable. In addition, tinnitus was defined as symptoms persisting for >6-months, which may have excluded acute or transient tinnitus cases; therefore, the null association observed for tinnitus should be interpreted cautiously. Another important limitation is that the DII was derived from 23 available dietary parameters in the KNHANES rather than the full 45 parameters originally proposed. Prior studies indicate that a modified DII including ≥ 20 key nutrients can still validly reflect the inflammatory potential of diet and has acceptable applicability in epidemiologic research. Nevertheless, the absence of certain components may lead to exposure misclassification, which could dilute the observed associations; therefore, the true associations may be underestimated. Although stratified analyses suggest a stronger association between DII and hearing loss as well as PV in older populations, the interaction between age and DII did not reach statistical significance, indicating insufficient evidence to support age as an effect modifier. Future studies could increase sample size and improve the representativeness of the older age group to verify whether age-related differences exist.

In conclusion, this study found that higher DII was associated with hearing loss and PV among Korean adults. Clinically, these findings support incorporating dietary assessment and nutrition counseling into comprehensive lifestyle management for individuals with auditory/vestibular symptoms. From a public health perspective, the present results add population-based evidence suggesting that pro-inflammatory dietary patterns may be associated with a higher burden of auditory and vestibular disorders, underscoring the potential value of dietary improvement as part of broader health-promotion strategies. However, given the cross-sectional design and the limitations of the dietary assessment, causal relationships and temporality cannot be determined. Future longitudinal and mechanistic studies are warranted to clarify the directionality of these associations and to explore whether reducing dietary inflammatory potential is associated with improved auditory and vestibular outcomes.

## Ethics approval and consent to participate

The KNHANES survey was approved by the Institutional Review Board of the Korea Centers for Disease Control and Prevention. As this study involved secondary analysis of publicly available data, no additional ethical review was required.

## Consent for publication

Not applicable.

## Disclosure of AI-assisted tools in manuscript preparation

During the preparation of this work, the author(s) used ChatGPT to assist with language editing and improving the clarity of the manuscript. After utilizing this tool, the author(s) thoroughly reviewed and edited the content as necessary and take(s) full responsibility for the final version of the manuscript.

## Authors' information

Not applicable.

## Funding

This study did not receive any funding support from the public, commercial, or non-profit sectors.

## CRediT authorship contribution statement

**Nan-Ren Sun:** Conceptualization, Methodology, Formal analysis, Visualization, Writing – original draft. **Sheng-Yuan Fang:** Data curation, Investigation, Software, Writing – review & editing. **Jiao-Jiao Chen:** Data curation, Investigation, Validation, Writing – review & editing. **Qingyang Wang:** Methodology, Resources, Writing – review & editing. **Zhi-Min Tan:** Conceptualization, Supervision, Project administration, Funding acquisition, Writing – review & editing.

## Declaration of competing interest

The author has no conflicts of interest to declare. The authors of this article do not report any financial disclosures.

## Data Availability

The datasets generated during and/or analyzed during the current study are available in the KNHANES database ().https://knhanes.kdca.go.kr/knhanes/main.do

## Appendix Supplementary materials

Image, application 1

## Footnotes

Supplementary material associated with this article can be found, in the online version, at doi:10.1016/j.clinsp.2026.101045.
