# Supplementary material for: The association of dietary inflammatory index with auditory and vestibular disorders in Korean adults: Differential links to peripheral vertigo, hearing loss and tinnitus
Source: Clinics (Sao Paulo). 2026 Jul 8;81:101045. doi: 10.1016/j.clinsp.2026.101045 (PMC13380207; doi:10.1016/j.clinsp.2026.101045)
Supplement: Supplementary file 1 [file mmc1.docx]

**CLINICS-D-25-02012_Supplementary Material**

**Supplementary Table S1** The 23 dietary components used to calculate the Dietary Inflammatory Index (DII).

| **Dataset variable** | **Standard name** |
| --- | --- |
| b_carotene | **Beta-carotene** |
| carbohydrates | **Carbohydrate** |
| cholesterol | **Cholesterol** |
| energy | **Energy** |
| fibre | **Dietary fiber** |
| folic_acid | **Folic acid** |
| Fe | **Iron** |
| Mg | **Magnesium** |
| MUFA | **Monounsaturated fatty acids** |
| niacin | **Niacin** |
| n-3_fatty_acids | **Omega-3 fatty acids** |
| n-6_fatty_acids | **Omega-6 fatty acids** |
| protein | **Protein** |
| PUFA | **Polyunsaturated fatty acids** |
| riboflavin | **Riboflavin (Vitamin B2)** |
| saturated_fat | **Saturated fatty acids** |
| tfat | **Total fat** |
| thiamin | **Thiamin (Vitamin B1)** |
| vitamin_A | **Vitamin A** |
| vitamin_C | **Vitamin C** |
| vitamin_D | **Vitamin D** |
| vitamin_E | **Vitamin E** |
| zinc | **Zinc** |

**Supplementary Table S2** Detailed sex-stratified subgroup analyses of associations between DII and Peripheral Vertigo (PV), overall hearing loss, and moderate-to-severe hearing loss (full covariate-level estimates).

| **Sex group / Co variate** | **Peripheral vertigo** | | **Moderate-to-severe hearing loss** | | **Overall hearing loss** | |
| --- | --- | --- | --- | --- | --- | --- |
|  | **Men** | **Women** | **Men** | **Women** | **Men** | **Women** |
| DII [G2 vs. G1] | 0.93 (0.74–1.17) | 1.06 (0.86–1.30) | 0.96 (0.76–1.22) | 1.13 (0.84–1.51) | 0.97 (0.81–1.17) | 0.95 (0.78–1.17) |
| DII [G3 vs. G1] | 0.93 (0.73–1.19) | 1.08 (0.88–1.32) | 1.18 (0.92–1.52) | 1.04 (0.79–1.38) | 1.10 (0.91–1.33) | 0.96 (0.79–1.16) |
| DII [G4 vs. G1] | 1.02 (0.79–1.33) | **1.29 (1.03–1.43)** | 1.08 (0.82–1.42) | **1.45 (1.11–1.89)** | 1.01 (0.81–1.25) | **1.21 (1.00–1.45)** |
| Residential area [G2 vs. G1] | 0.99 (0.81–1.22) | **1.21 (1.03–1.43)** | 0.88 (0.72–1.09) | 1.18 (0.96–1.45) | **1.27 (1.07–1.50)** | **1.33 (1.14–1.55)** |
| Household income | **0.91 (0.84–0.99)** | 0.95 (0.89–1.01) | 0.94 (0.86–1.02) | **0.89 (0.81–0.97)** | 1.05 (0.98–1.12) | 1.05 (0.99–1.12) |
| Education [G2 vs. G1] | 1.02 (0.83–1.25) | 1.12 (0.94–1.33) | **0.63 (0.50–0.81)** | **0.59 (0.42–0.83)** | **0,30 (0,26–0.35)** | **0.24 (0.20–0.28)** |
| Occupation [G2 vs. G1] | 0.96 (0.79–1.17) | 0.92 (0.77–1.10) | 0.98 (0.80–1.18) | 0.98 (0.80–1.21) | **0.82 (0.70–0.96)** | 1.14 (0.98–1.34) |
| BMI [G1 vs. G2] | 0.85 (0.49–1.45) | 0.90 (0.62–1.30) | 0.99 (0.58–1.68) | 1.12 (0.69–1.83) | 1.52 (0.91–2.54) | 1.11 (0.76–1.62) |
| BMI [G3 vs. G2] | 1.02 (0.84–1.24) | 0.92 (0.79–1.06) | 0.98 (0.81–1.19) | 0.96 (0.79–1.17) | **0.68 (0.59–0.79)** | 0.97 (0.84–1.12) |
| Drinking status [G2 vs. G1] | 1.08 (0.68–1.71) | 0.97 (0.80–1.16) | 0.86 (0.56–1.30) | 0.99 (0.81–1.22) | **0.41 (0.29–0.59)** | **0.48 (0.41–0.56)** |
| Marital [G2 vs. G1] | 1.12 (0.80–1.58) | 1.01 (0.70–1.45) | 1.15 (0.71–1.87) | 1.37 (0.71–2.65) | **0.40 (0.30–0.53)** | 0.87 (0.55–1.37) |
| Walking training [G2 vs. G1] | 1.06 (0.82–1.37) | 0.96 (0.81–1.23) | **0.75 (0.58–0.97)** | 0.93 (0.72–1.20) | **0.82 (0.67–1.00)** | 0.85 (0.70–1.02) |
| Walking training [G3 vs. G1] | 1.22 (0.95–1.57) | 1.10 (0.90–1.34) | 0.85 (0.66–1.10) | 0.86 (0.67–1.10) | 1.07 (0.87–1.30) | 0.84 (0.70–1.01) |
| Weight training [G2 vs. G1] | 1.11 (0.88–1.40) | 0.97 (0.78–1.18) | 0.89 (0.69–1.14) | 0.97 (0.68–1.38) | 0.84 (0.69–1.01) | 0.93 (0.74–1.16) |
| Weight training [G3 vs. G1] | 0.80 (0.60–1.08) | 0.95 (0.73–1.25) | 0.79 (0.61–1.01) | 1.01 (0.72–1.42) | 1.09 (0.89–1.34) | 1.24 (0.96–1.60) |
| Smoking status [G2 vs. G1] | 0.90 (0.69–1.19) | 1.19 (0.84–1.69) | 1.28 (0.96–1.69) | 0.80 (0.42–1.51) | **1.51 (1.19–1.93)** | 1.03 (0.70–1.52) |
| Smoking status [G3 vs. G1] | 0.87 (0.68–1.18) | 1.10 (0.83–1.69) | 1.16 (0.92–1.48) | 1.22 (0.78–1.91) | 1.20 (0.97–1.50) | 0.96 (0.68–1.34) |
| Psychological stress [G1 vs. G4] | **3.64 (2.27–5.84)** | **2.30 (1.63–3.27)** | 0.97 (0.57–1.64) | 0.76 (0.46–1.27) | 0.87 (0.52–1.46) | 1.05 (0.70–1.57) |
| Psychological stress [G2 vs. G4] | **2.54 (1.87–3.45)** | **1.91 (1.53–2.39)** | 1.01 (0.75–1.37) | 0.99 (0.74–1.31) | 0.86 (0.65–1.13) | 0.96 (0.76–1.23) |
| Psychological stress [G3 vs. G4] | **1.54 (1.19–1.99)** | **1.36 (1.13–1.65)** | 0.95 (0.78–1.17) | 1.11 (0.89–1.39) | 0.96 (0.78–1.19) | 1.04 (0.86–1.26) |
| Hypertension [G2 vs. G1] | **1.23 (1.00–1.50)** | 1.10 (0.94–1.30) | 1.06 (0.88–1.28) | 1.11 (0.91–1.35) | **1.86 (1.60–2.16)** | **2.27 (1.98–2.61)** |
| Diabetes [G2 vs. G1] | 1.19 (0.94–1.51) | 0.97 (0.78–1.21) | 0.93 (0.76–1.14) | 1.18 (0.94–1.49) | **1.36 (1.13–1.64)** | **1.78 (1.47–2.15)** |

**Supplementary Table S3** Detailed age-stratified subgroup analyses of associations between DII and Peripheral Vertigo (PV), overall hearing loss, and moderate-to-severe hearing loss (full covariate-level estimates).

| **Age group / Co variate** | **Peripheral vertigo** | | **Moderate-to-severe hearing loss** | | **Overall hearing loss** | |
| --- | --- | --- | --- | --- | --- | --- |
|  | **Low Age (≤60)** | **High Age (>60)** | **Low Age (≤60)** | **High Age (>60)** | **Low Age (≤60)** | **High Age (>60)** |
| DII [G2 vs. G1] | 1.03 (0.83–1.28) | 0.93 (0.75–1.15) | 0.96 (0.63–1.46) | 1.12 (0.92–1.35) | 0.95 (0.76–1.18) | 1.12 (0.93–1.34) |
| DII [G3 vs. G1] | 1.03 (0.83–1.28) | 0.97 (0.78–1.20) | 1.11 (0.73–1.68) | **1.22 (1.01–1.48)** | 1.01 (0.81–1.27) | **1.21 (1.01–1.44)** |
| DII [G4 vs. G1] | 1.11 (0.90–1.38) | **1.28 (1.04–1.58)** | 1.12 (0.75–1.67) | **1.52 (1.25–1.85)** | 1.02 (0.81–1.28) | **1.49 (1.23–1.81)** |
| Residential area [G2 vs. G1] | 1.14 (0.93–1.39) | 1.08 (0.92–1.28) | 1.01 (0.70–1.44) | 1.09 (0.94–1.27) | 1.13 (0.93–1.38) | **1.18 (1.02–1.38)** |
| Household income | 0.99 (0.81–1.21) | **0.93 (0.87–1.00)** | 0.94 (0.83–1.07) | 0.96 (0.90–1.02) | 1.03 (0.96–1.11) | 0.98 (0.93–1.05) |
| Education [G2 vs. G1] | 0.83 (0.53–1.29) | 0.93 (0.74–1.16) | **0.35 (0.25–0.49)** | **0.59 (0.48–0.73)** | **0.43 (0.36–0.51)** | **0.55 (0.45–0.66)** |
| Occupation [G2 vs. G1] | 0.97 (0.82–1.15) | 0.89 (0.75–1.05) | 1.25 (0.92–1.70) | **0.78 (0.67–0.91)** | 1.09 (0.91–1.32) | 0.90 (0.77–1.04) |
| BMI [G1 vs. G2] | 0.89 (0.53–1.49) | 0.99 (0.66–1.48) | 0.62 (0.23–1.66) | 1.23 (0.85–1.77) | 0.95 (0.54–1.66) | 1.16 (0.75–1.78) |
| BMI [G3 vs. G2] | 0.88 (0.69–1.11) | 0.90 (0.77–1.06) | 0.85 (0.62–1.17) | **0.85 (0.74–0.98)** | 0.94 (0.79–1.11) | 0.94 (0.82–1.07) |
| Drinking status [G2 vs. G1] | 0.86 (0.62–1.19) | 1.00 (0.83–1.21) | **0.47 (0.27–0.83)** | **0.77 (0.65–0.92)** | **0.53 (0.39–0.73)** | **0.79 (0.67–0.94)** |
| Marital [G2 vs. G1] | 1.08 (0.83–1.41) | 0.87 (0.48–1.57) | 0.72 (0.44–1.19) | 1.07 (0.60–1.91) | **0.70 (0.53–0.93)** | 0.88 (0.49–1.56) |
| Walking training [G2 vs. G1] | 1.12 (0.89–1.42) | 0.92 (0.74–1.14) | 0.84 (0.56–1.25) | **0.76 (0.63–0.93)** | 0.89 (0.71–1.11) | 0.85 (0.69–1.04) |
| Walking training [G3 vs. G1] | **1.33 (1.05–1.68)** | 0.95 (0.78–1.16) | 1.02 (0.68–1.52) | **0.78 (0.65–0.94)** | 0.90 (0.72–1.23) | 0.85 (0.70–1.03) |
| Weight training [G2 vs. G1] | 1.08 (0.88–1.32) | 0.97 (0.77–1.22) | 1.09 (0.74–1.61) | **0.72 (0.57–0.89)** | 0.96 (0.78–1.20) | **0.78 (0.64–0.96)** |
| Weight training [G3 vs. G1] | 1.10 (0.81–1.50) | **0.75 (0.59–0.96)** | 0.89 (0.52–1.51) | 0.93 (0.76–1.14) | 0.99 (0.74–1.32) | 0.98 (0.80–1.20) |
| Smoking status [G2 vs. G1] | 0.97 (0.74–1.27) | 1.04 (0.77–1.40) | 1.44 (0.89–2.32) | 0.79 (0.62–1.02) | **1.40 (1.09–1.81)** | 0.98 (0.74–1.30) |
| Smoking status [G3 vs. G1] | 0.92 (0.72–1.17) | 1.09 (0.84–1.42) | **1.60 (1.04–2.48)** | 1.00 (0.81–1.23) | **1.27 (1.01–1.59)** | 0.97 (0.77–1.21) |
| Psychological stress [G1 vs. G4] | **3.02 (2.05–4.45)** | **1.95 (1.30–2.93)** | 0.60 (0.23–1.61) | 0.79 (0.52–1.18) | 0.81 (0.52–1.24) | 0.70 (0.46–1.05) |
| Psychological stress [G2 vs. G4] | **2.02 (1.54–2.65)** | **2.07 (1.63–2.64)** | 0.98 (0.58–1.64) | 0.81 (0.65–1.01) | **0.66 (0.50–0.86)** | **0.77 (0.62–0.96)** |
| Psychological stress [G3 vs. G4] | **1.28 (1.00–1.65)** | **1.53 (1.27–1.84)** | 1.39 (0.88–2.20) | **0.77 (0.66–0.89)** | 0.86 (0.68–1.08) | **0.83 (0.71–0.96)** |
| Hypertension [G2 vs. G1] | 1.13 (0.91–1.39) | **1.29 (1.11–1.50)** | 1.22 (0.86–1.72) | **1.45 (1.26–1.66)** | **1.34 (1.10–1.63)** | **1.39 (1.21–1.59)** |
| Diabetes [G2 vs. G1] | **1.61 (1.22–2.12)** | 0.84 (0.70–1.01) | 0.72 (0.44–1.19) | 1.14 (0.98–1.34) | 1.13 (0.87–1.46) | **1.35 (1.14–1.59)** |
